# Effects of aligning prisms on the objective and subjective fixation disparity in far distance

**DOI:** 10.16910/jemr.12.4.8

**Published:** 2019-12-11

**Authors:** Volkhard Schroth, Roland Joos, Ewald Alshuth, Wolfgang Jaschinski

**Affiliations:** 1 FHNW, Institute of Optometry, Olten, Switzerland; 2 Leibniz Institute for Working Environment and Human Factors, Dortmund, Germany; 3 Hagen, Germany

**Keywords:** Eye tracking, fixation disparity, vergence, aligning prism, Cross test, MCH-procedure

## Abstract

Fixation disparity (FD) refers to a suboptimal condition of binocular vision. The oculomotor aspect of FD refers to a misadjustment in the vergence angle between the two visual axes that is measured in research with eye trackers (objective fixation disparity, oFD). The sensory aspect is psychophysically tested using dichoptic nonius lines (subjective fixation disparity, sFD). Some optometrists use nonius tests to determine the prisms for constant wear aiming to align the eyes. However, they do not (yet) use eye trackers. We investigate the effect of aligning prisms on oFD and sFD for 60 sec exposure duration of prisms determined with the clinically established Cross test in far distance vision. Without prisms, both types of FD were correlated with the aligning prism, while with prisms the FD was close to zero (these analyses included all base-in and base-out cases). The effect of base-in prisms on oFD was proportional to the amount of the aligning prism for the present 60 sec exposure, similar as for the 2- 5 sec exposure in Schmid et al. (2018). Thus, within 1 minute of prism exposure, no substantial vergence adaptation seems to occur in the present test conditions. Further studies may investigate intra-individual responses to different exposure times of aligning prisms in both prism directions.

## Introduction

The two eyes in humans provide advantages such as binocular summation and depth perception [1-3]. However, in individuals with suboptimal coordination of the two eyes, disadvantages can occur, e.g. visual complaints or even diplopia can occur in extreme cases. In diplopia, the perceived offset of the two images indicate a misadjustment of the vergence angle between the two visual axes. More generally, the perceived angular offset between non-fused images in the two eyes quantifies this misadjustment. Accordingly, two non-fusible test targets in both eyes are applied in clinical optometry as a tool to measure the vergence angle. Such methods are referred to as subjective since they rely on the subject’s perception. E. g., for measuring heterophoria (the fusion free vergence state), the so-called Maddox-wing test presents a horizontal scale to the left eye and an arrow to the right eye, when no fusion stimulus is present [4]. Test results of fusion-free vergence were shown to agree with results of objective tests based on physical instrumentation as eye trackers [5-8].

For many decades, subjective test devices have also been used with the aim of measuring vergence in the condition of fusion: a binocularly visible target is superimposed by non-fusible nonius lines that were dichoptically presented to the right and left eye [9-11]. When shifting the nonius lines relative to each other until they appear in alignment, the resulting nonius offset provides the subjective fixation disparity with the angular unit “minutes of arc” [12-14]. The latter procedure can be used with devices that allow to shift the nonius lines. Most clinical tests such as the Mallett-unit [15] or the MCH-tests [16], however, use dichoptical nonius targets that are presented in physical alignment and the optometrist then determines the individual amount of “aligning prism” with which the patient perceives the nonius lines in alignment [9]. The power of the prism is indicated by the unit “prism dioptre (pdpt)” or “cm/m”. It is important to note that there is no fixed geometrical relation between the subjective fixation disparity and the aligning prism, although both measures are based on the same perceived test target. This is because the functional relation between the applied prism and the fixation disparity (known as “fixation disparity curve”) depends on the individual [11, 17, 18].

For a long time, the subjective fixation disparity as measured with dichoptic nonius lines was understood as a measure of the vergence error and was referred to as fixation disparity. Since the 1980s, however, objective measurements of vergence errors with high-resolution eye tracking devices have shown a clear difference between the subjective and objective measures of fixation disparity [19-21]. The objective fixation disparity is a deviation of the current vergence angle from the vergence stimulus; objective fixation disparity is measured as the deviation of the prevailing vergence angle from the optimal vergence angle; the zero eye position is calibrated by means of monocular fixation targets.

Thus, objective fixation disparity reflects the oculomotor vergence error, while the subjective fixation disparity includes sensory/neural processes, which facilitate fusion despite a motor vergence error [17, 22].

A fixation disparity is a sub-optimal state of binocular vision that one may wish to compensate by wearing appropriate eye glasses. This is possible with prism eye glasses since the state of fixation disparity depends on the amount and direction of a prism [11]. Some approaches in optometry suggest wearing prism eye glasses to reduce fixation disparity to zero aiming to reduce asthenopic complaints; examples are the application of the Mallett-unit [9, 23], or the MCH-procedure [24, 25].

Reducing the fixation disparity to zero seems to be straight forward but it is complicated by the following discrepancy. In clinical optometry, technically simple test devices with dichoptic nonius tests are used to subjectively determine the the fixation disparity and the aligning prism [9, 10, 26-28]. The objective fixation disparity requires elaborate eye tracker procedures that cannot be applied in clinical optometry and are therefore limited to the research laboratory. The relationship between these subjective and objective measures has just begun to be studied.

Measurements of both subjective and objective fixation disparity in the context of aligning prisms intended for clinical purposes have only been applied in two earlier studies [29, 30].

**Figure 1:**
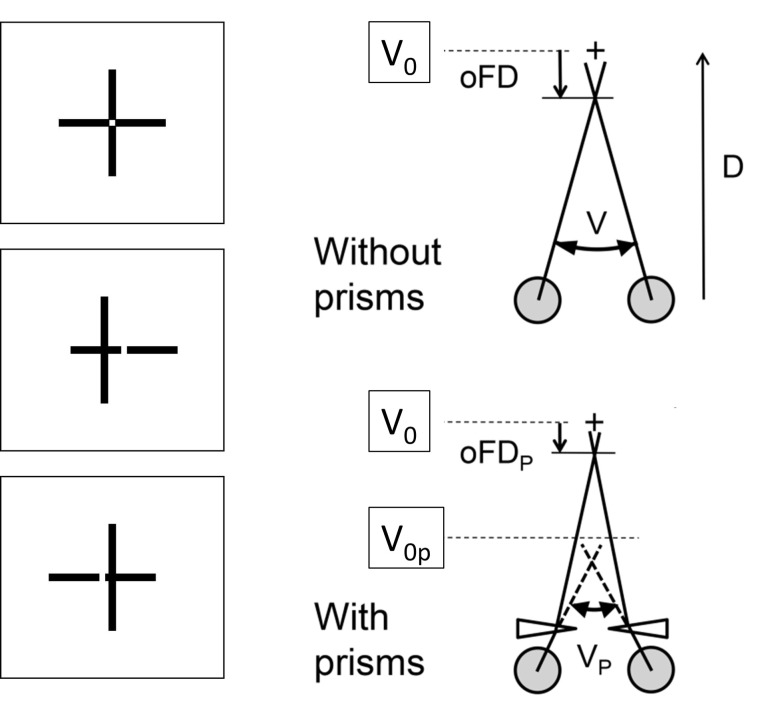
(Left) Cross test with the vertical and horizontal lines in alignment and with an offset to the left and to the right. The angular amount of the offset at perceived alignment represents the subjective fixation disparity. (Right) The eye tracker measures the vergence angle V between the visual axes. The deviation of V from the stimulus vergence angle V_0_ is the objective fixation disparity. The prism introduces a shift of the vergence stimulus from V_0_ to V_0p_. The angles are not to scale. For more details see Schroth et al. [29].

In the earlier study, Schroth et al. [29] showed the expected reduction in subjective and objective fixation disparity due to the aligning prisms at least under some measurement conditions; however, the prism effect can be different for the two types of fixation disparity. It may depend on the prism direction (base-in versus base-out) and on individual vergence parameters. Schroth et al. [29] used an individual amount of the aligning prism which resulted from the so-called MCH-procedure that included a series of different nonius type of tests: a test with only a peripheral fusion target (Cross test; Figure 1) is mainly intended to correct the motor aspect of fixation disparity; additional tests with central fusion targets intend to also reveal the sensory aspect of fixation disparity. Schroth et al. had the participants wear the prism spectacles for about 5 weeks before the prism effect was measured. Thus, the Schroth et al. [29] study mimicked the prescription of prisms in optometric practice.

Schmid et al. [30] determined the aligning prism with the Cross test. Repetitive recordings for 2 – 5 seconds were made over a 1 minute period with and without this aligning prism. This test condition is recommended in clinical optometry to avoid vergence adaptation: the subjective and objective prism effects on sFD and oFD were correlated with the amount of the aligning prism, at least for base-in cases.

These two earlier studies differed (1) the test target used to determine the aligning prism and they differed (2) considerably in the prism exposure duration.

The test target for the aligning prism in the study of Schmid et al. [30] was the Cross test that does not include a central fusion target, whereas Schroth et al. [29] included further tests with central fusion stimuli. The results based on tests with only peripheral fusion targets should predominantly reflect the oculomotor vergence state [20] and, therefore, the objective fixation disparity should be well predicted by the aligning prism based on a Cross test. In order to facilitate the interpretation, the present study uses the Cross test

The prism exposure duration is relevant for the potential adaptation of the vergence system. The vergence angle is modified over time according to the prevailing vergence demand, which is given by the current viewing distance in natural vision. In optometric testing, the vergence demand can be modified by prisms in front of the eyes. The classical and well investigated indicator used for measuring vergence adaptation is heterophoria, i.e. the vergence angle without a fusion target [31-34]. Fewer studies refer to the condition of fusion [35, 36]. The typical approach is to measure the initial fixation disparity without prisms, then to place prisms in front of the eyes for different periods of time and to subsequently measure the fixation disparity again. For binocular vision diagnoses based on fixation disparity curves [11, 37, 38], the optometrist wishes to avoid vergence adaption and therefore short prism exposures of only few seconds are applied [39]; resulting in large changes in fixation disparity – depending on the individual. However, the longer the prisms are applied the larger is the adaptation effect. If the optometrist wishes to prescribe aligning prisms for constant wear, it may be questionable whether a sustained change in fixation disparity can found or whether vergence adaption has restored the initial fixation disparity again.

Our two earlier studies had used extremely different prism exposure durations of 6 weeks [29] and 2 – 5 seconds [30]. The present study used an intermediate prism exposure duration of 1 minute that seemed to be a reasonable choice for two reasons. First, Howard summarized the studies of Schor [1, 40, 41] as follows: “fixation disparity and phoria can begin to change within the first minute of exposure to base-out or base-in prisms”. Second, a period of one minute is not too long, thereby, reliable measure of the technically difficult recording of objective fixation disparity can be made, as we found in our earlier studies.

Thus, the present study addresses the following three main topics:

1. In natural vision (i. e. no prisms) the regression is tested how the individual aligning prism is able to predict
  - The subjective fixation disparity; given that both these measures are subjective and rely on the same test target, a regression should be expected, but the quantitative relation was not yet investigated for the present Cross test.
  - The objective fixation disparity, since users of the Cross test assume that the corresponding aligning prism should reflect the oculomotor vergence error (oFD). But this has not yet been confirmed experimentally.
2. The conditions without and with prisms are compared regarding how the individual amount of the aligning prism is able to correct the subjective and objective fixation disparity.
3. The individual aligning prism is tested to see how it is able to predict the change both in subjective and in objective fixation disparity.

## Methods

The methods are similar to those used in our previous study [29]. Stimuli appeared at 5 m viewing distance on a 3D-television monitor (LG 32 LW 4500), subtending a visual angle of 6.6 deg horizontally and 3.7 deg vertically. The 3D-mode was required in order to use dichoptic nonius targets to measure subjective fixation disparity (sFD). Eye movements were measured with the EyeLink II system (SR Research), however in a modified way for the precise recording of the small amount of objective fixation disparity (oFD) below 2 deg [7].

### Stimuli and apparatus

Figure 1 illustrates the structure and the dimensions of the stimulus that comprised a central cross with dichoptic nonius lines and a peripheral quadratic frame with horizontal fusion contours at ± 1.6 deg. The luminance was 90 cd/m^2^ in the square and 20 cd/ m^2^ on the screen background, as measured through the polarizers.

Subjective fixation disparity was measured using central dichoptic nonius targets, i.e., a pair of vertical lines (each 0.6± long, 0.07± wide) was visible for the right eye and a pair of horizontal lines (each 0.6± long, 0.07± wide) was visible for the left eye. Both pairs of lines had a central gap of 0.07±; see Figure 1 for misaligned and aligned conditions. The observer adjusted the lines to alignment. This Cross test was proposed as part of the “Measuring and Correcting Methodology after H.-J. Haase (MCH)” [16, 28, 42]. This methodology suggests prisms for constant wear to reduce asthenopic complaints [43]. In the present MCH-procedure, the aligning prism is determined predominantly by a far-vision test (although a near-vision test is also available); still, clinical experience showed that near vision complaints are reduced [16, 24]. The Cross test is the first in the series of MCH-tests and is supposed to predominantly identify the oculomotor component of fixation disparity [44]. The repeatability of MCH tests was investigated by Alhassan et al. [42]. For the Cross test in far distance, the 95% confidence intervall of agreement was in the range from -0.88 to 0.75 pdpt if the subjects were asymptomatic. This could be stated as a good repeatability with a standard deviation of 0.4 pdpt.

A single recording for a one-minute data collection was made as follows: In a series of nonius adjustments, the observer shifted the vertical lines to a perceived alignment relative to the gap between the horizontal lines by using the left and right button of the computer mouse. Once the subjective alignment was reached, the observer clicked the centre computer mouse button. The resulting nonius offset was recorded as a single data point of the subjective fixation disparity and the corresponding eye position and pupil size were determined as follows: The median and standard deviation of the objective fixation disparity was calculated offline across the interval 100 to 400 ms before clicking; this period was chosen to reduce artefacts due to blinking at the moment of clicking. The median of these standard deviations was 2.1 min arc. In 3% of all single data points the standard deviation was larger than 10 min arc; these single data points then were discarded. One nonius adjustment took only a few seconds.

### Eye movements recordings

The video-based EyeLink II (SR Research Ltd, Osgoode ON, Canada) was used with the dark pupil detection mechanism that tracks the centre of the pupil. Recorded data were analyzed based on the raw data, which were sampled every 2 ms (500 Hz). The filters of the EyeLink software were switched off. The conventional EyeLink II procedures were modified in order to improve the measuring performance for fixation disparity; the accuracy of the present recording and the measurement approach are described fully in Jaschinski [45] and Schroth et al. [29]. In short, the recording system has a physical resolution of 0.6 min arc. In order to reduce errors introduced due to calibration and during the recording process, the present procedure used a short 1-minute recording period with a pre- and a post calibration, a rigid head stabilization, and a series of repeated measurements that were averaged to reduce random error. Instead of the original EyeLink II calibration mode, we used the raw data and applied the following monocular calibrations before and after the 1-minute recording period that were then averaged. The use of polarizors is not sufficient for complete monocular vision during the calibration since the mechanical frame of the display can be effective as a peripheral fusion target. Therefore, the right eye was covered with a purpose made opaque occluder to calibrate the left eye and, subsequently, the left eye was covered to calibrate the right eye. The opaque occluder was chosen to make all stimuli invisible, but also to lower the luminance by only 30% so that the pupil would only slightly dilate due to the occlusion. For the calibration, subjects were requested to carefully fixate on one of three calibration targets (crosses of 14 min arc) that appeared sequentially in the screen centre (zero position) on the left and right horizontal positions of 2.3 deg. Each of the three calibration targets was presented twice randomly to average across variability in fixation.

### Design of the study.

Data collection was performed for each subject during two experimental sessions on different days and it took about 30 minutes each day. Day 1 included a monocular, optometric refraction and a determination of the aligning prisms at the Cross test in accordance with the IVBS guidelines [46]. Day 2 comprised of eight eye tracking recordings (each lasted one minute plus a pre- and a post calibration) that were made alternately without and with the aligning prism. The series always started without a prism.

### Participants

The sample of 16 subjects was selected for a visual acuity ≥ 0.8 (decimal units) without any optical correction since spectacles can prevent precise eye movement recordings. Exclusion criteria were nystagmus and strabismus tested using the unilateral cover test. Two subjects were excluded because the aligning prism was at zero, another subject was excluded because on Day 2 he was not able to fuse the test figure with his large base-out prism. The database for the statistics comprised of 13 subjects whose age was 24.7 ± 2.3 years (mean ± SD). The ametropia did not exceed ± 0.75 dpt (sph-cyl equivalent). This research was approved by the Ethics Committee Northwestern Switzerland (EKNZ). The procedures were in accordance with good clinical practice and participants signed a written informed consent.

### Data analysis and statistics

Regression lines are used to analyze how the vergence error (objective fixation disparity, oFD) and the nonius offset (subjective fixation disparity, sFD) depend on the amount of the aligning prism determined at the Cross test. The analyses and the graphs were made with the open-source software R. Robust regressions were calculated with the procedure lmrob from the package robustbase, in which outlier data points are weighted less [47]. This regression analysis provides the coefficients of linear equations, their standard errors SE, t-statistics and probabilities; 1.96 times the SE is half the width of the confidence interval CI of these coefficients. If the half of the width of the CI, subsequently abbreviated as HWCI, is smaller than the amount of the coefficient, the coefficient will differ significantly from zero (p < 0.05). Furthermore, lmrob gives the adjusted R squared: this percentage indicats the extent to which the inter-individual variability of fixation disparity can be explained by the inter-individual variation of the aligning prism.

Recordings of video eye trackers can be contaminated by a specific physiological pupil effect: the center of the pupil typically shifts towards the nose when the pupil shrinks. This will appear as an artefactual nasal eye movement by video eye trackers that are based on the center of the pupil. In the present study, this artifact was corrected using a procedure described by Jaschinski [48]. Since the center of the pupil can shift laterally when the pupil size changes (for whatever reason), the eye tracker calibration is only valid, if the pupil size during the recording P_rec_ is identical to the pupil size during the calibration P_cal_. Therefore, a linear regression of single oFD-measure as a function of corresponding pupil sizes P_rec_ was calculated for all data within a 1-minute recording period. From this equation, the predicted oFD-value corresponding to P_rec_ = P_cal_ was calculated.

## Results

The results are presented according to the three main topics of this study as stated in the Introduction.

**Figure 2:**
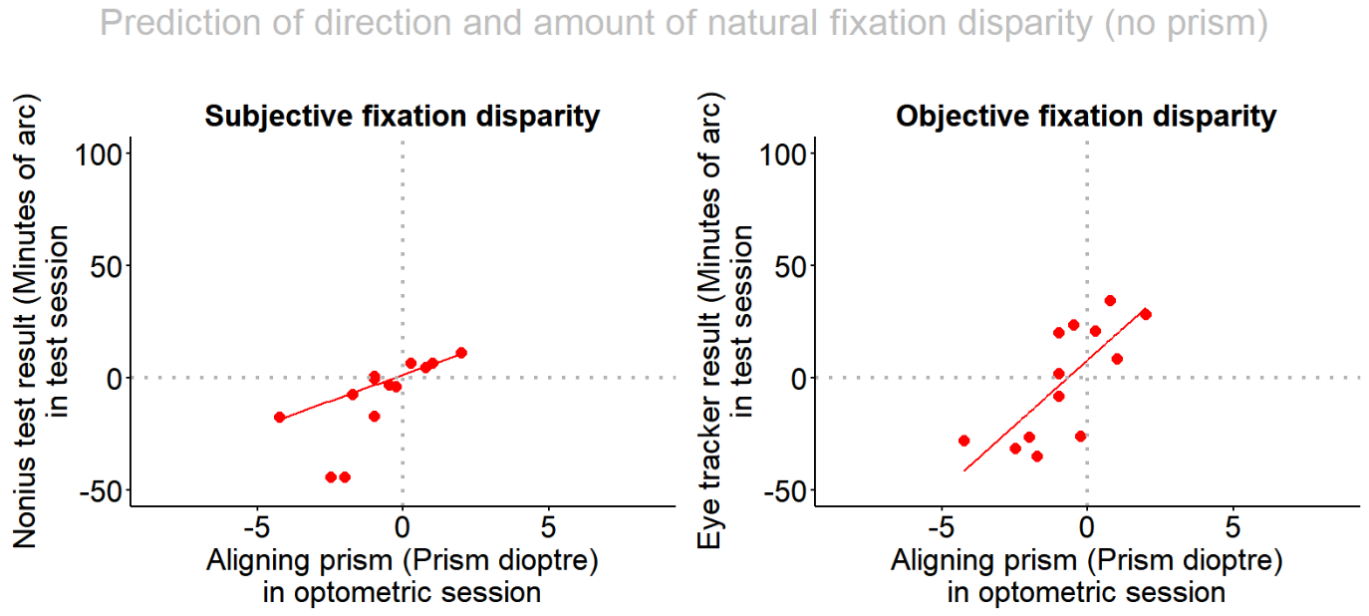
Subjective and objective fixation disparity (measured without wearing aligning prisms) depending on the aligning prism. The fixation disparity has a negative sign for exo, under-convergent states and a positive sign for eso, over-convergent states. The aligning prism has a positive sign for base-out prisms and a negative sign for base-in prisms. All 13 subjects were included in this robust regression analysis.

Figure 2 shows the results when no prism was applied during the eye tracker recording. This analysis answers the question if the direction and the amount of a naturally occurring objective fixation disparity (without wearing prisms) can be predicted by the aligning prism determined with the clinically applied subjective test of fixation disparity using dichoptic nonius targets. For subjects with base-in aligning prisms (negative sign), one expects a vergence error in the exo direction (negative sign), while base-out prisms (positive sign) should correspond to eso vergence errors (positive sign); moreover, the amount of fixation disparity should increase with the amount of the aligning prism.

The regression equation for objective fixation disparity without prisms (oFD_0_) showed a significant slope: oFD_0_ = 8.19 _12.05_ + P * 11.71 6.80; adjusted R-squared 0.49; p = 0.008; the subscripted values indicate half of the width of the 95%-confidence interval of each coefficient (HWCI). The HWCI of 6.80 is smaller than the slope of 11.71, thus the effect of the amount of the prism is statistically significant. The slope of the regression line indicates that a change in the aligning prism by one prism dioptre corresponds to a change in the objective fixation disparity by 11.71 min arc. For 10 of the 13 subjects the data points are in the first and third quadrant, as should be expected.

For subjective fixation disparity, all data points lay in the hypothesized first and third quadrant. The regression without wearing the prisms (sFD_0_) has a significant slope: sFD_0_ = 1.53 _2.07_ + P * 4.62 _1.22_ ; adjusted R-squared 0.80; p < 0.0001. This is not surprising since the aligning prism had been determined by the subjective Cross test. The slope of the regression line suggests that a one-prism diopter change in the aligning prism corresponds to a change in subjective fixation disparity by 4.61 min arc.

**Figure 3:**
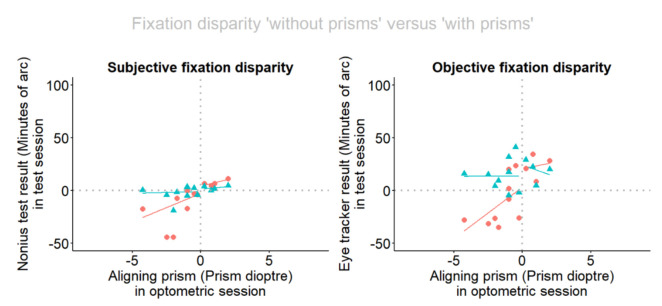
Fixation disparity depending on the aligning prism. Red dots and lines refer to the situation without prism, blue triangles and lines refer to the situation when prisms are worn. The fixation disparity has a negative sign for exo, under-convergent states and a positive sign for eso, over-convergent states. The aligning prism has a positive sign for base-out prisms and a negative sign for base-in prisms.. (a) Subjective fixation disparity is given by the nonius offset of the dichoptic targets and (b) objective fixation disparity is given by the eye recording of the vergence error. The robust regression lines refer to separate analyses for base-in and base-out cases.

Figure 3 provides an overview of the conditions without (red dots) and with prisms (blue triangles) in relation to the amount of the aligning prism, for each type of fixation disparity. The regression lines are shown separately for the base-in (n = 9) and for the base-out (n = 4) cases since our previous work has shown that prism effects can differ between these two directions of fixation disparity [29, 36]. Note that the individual amount of the prism was determined in Session 1 and that in Session 2 recordings of subjective and objective fixation disparity were made alternating between without and with these prisms.

The following statistical analyses are confined to the larger of the two subgroups, i. e. the 9 base-in cases; the smaller subgroup of 4 base-out cases is too small to perform convincing statistics.

Without prisms the regressions lines are: sFD_0_ = -3.74 _21.67_ + P * 7.102 _11.05_; adjusted R-squared 0.12; oFD_0_ = 3.37 _25.81_ + P * 9.84 _13.1_; adjusted R-squared 0.15. Both are not significant, presumably because of the smaller size of the subsamples compared to the complete sample. However, the insignificant trend in Figure 3 for the sub-sample resembles the significant regression in the complete sample shown in Figure 2.

When wearing the prisms, sFD is shifted in the eso direction in all base-in subjects and the blue regression line suggests that sFD_P_ is close to zero and does not depend on the amount of the aligning prism. Accordingly, the regression line of sFD_P_ is not significant: sFD_P_ = -1.25 _6.05_ + P * 0.37 _3.85_; adjusted R-squared 0.0. The aligning prism also shifted the oFD in the expected positive direction. As expected, the amount of the aligning prism had no effect on objective fixation disparity, when the prism was worn: oFD_P_ = 14.56 _19.96_ + P * 0.42 _10.10_; adjusted R-squared 0.0. The resulting level of about 15 min arc was not significant in the positive range

**Figure 4:**
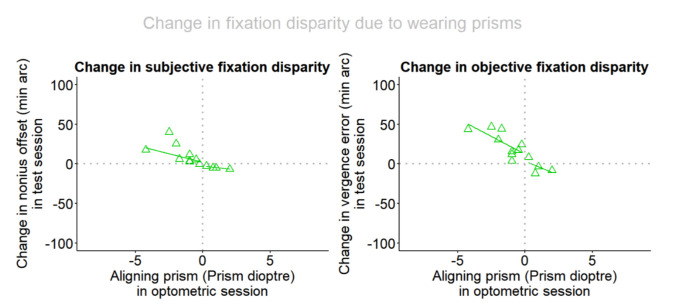
The change in subjective and objective fixation disparity as a function of the amount of the aligning prism. Separate robust regression lines are included for base-in and base-out cases.

In addidion to the presentation of the fixation disparity in the two prism conditions in Figure 3, the change induced by the prisms was calculated as the difference “with prism” minus “without prism” and is plotted in Figure 4. The reason being that the calculation of objective fixation disparity includes an uncertainty which is related to the definition of the individual zero-condition of objective fixation disparity and the calibration of the eye tracker [17]. This caveat does not play a role when the difference between two corresponding measures of objective fixation disparity are considered. Accordingly, Figure 4 shows the data points of these changes. Again, the regression lines are shown separately for the base-in and the base-out cases and the statistics are confined to the 9 base-in cases. For objective fixation disparity, the regression of the changes confirm a linear relation to the amount of the aligning prism with a significant slope: oFD_δ_ = 12.11 _14.50_ - P * 9.08 _7.36_; adjusted R-squared: 0.40; p = 0.034. For subjective fixation disparity, the linear regression does not reach significance, but a trend does appear: sFD_δ_ = 2.15 _13.52_ - P * 6.07 _6.96_; adjusted R-squared 0.25; p = 0.087.

## Discussion

The discussion follows the three research questions described in the Introduction.

It was first questioned whether the direction and the amount of the individual aligning prism is able to predict the objectively measured fixation disparity when no prism is worn by the subject. Historically, optometrists use the aligning prisms (determined at the subjective Cross test) as an estimation of the motor vergence position, however optometrists cannot measure the oculomotor vergence position objectively since the required sophisticated eye tracker procedures are beyond the scope of a clinical setting. The reported significant correlation and the regression line suggest that a prediction is possible to the extent that about 50% of the inter-individual variability in objective fixation disparity can be explained by the subjectively determined aligning prism. Schmid et al. [30] did a very similar experiment (also using the Cross test): here we did an additional regression analysis of their published data: oFD_0_ = 13.46 _13.21_ + P * 6.18 _4.77_ ; adjusted R-squared 0.40; p = 0.025. Schroth et al. [29] applied an aligning prism based on the Cross test and did further additional tests that included central fusion stimuli: the resulting correlation with the objective fixation disparity was: oFD_0_ = 14.38 _8.65_ + P * 4.22 _2.25_; adjusted R-squared 0.38; p = 0.016. It appears that per one prisms diopter of the individual aligning prism the objective fixation disparity (recorded without wearing the prism) changes by 4.62, 6.18, and 4.22 min arc in the present study, in Schmid et al. [30] and in Schroth et al. [29], respectively. Regarding the HWCI (indicated as subscripts) these regression slopes are somewhere between these three studies, despite the methodological differences mentioned above. Note that this interpretation is based on regression lines across different observers and that the slope describes the average effect of an individual aligning prism in a group of subjects. Another and more common analysis is the effect of the variation of the amount of the experimental prism within a single observer, known as “fixation disparity curves” [11, 17].

The second research question specifically addressed the subgroup of 9 subjects who required base-in prisms. The reason was twofold: (First) base-in and base-out cases may differ due to the nature of prism effects [29] and (Second) the present subsample of only 4 base-out cases was too small for statistical analyses. Accordingly, we conclude that the expected effect of the prisms was confirmed because in all cases with base-in prisms the fixation disparity was shifted in the eso direction. In subjective fixation disparity, the corrected fixation disparity was very close to zero; this is self-evident since the prism was determined based on a subjective judgement at the Cross test. More interesting was the objective fixation disparity shift in the eso direction in all cases. However, the resulting level was not zero, but positive on average at a level of about 15 min arc, however, this is not significantly different from zero, since the y-intercept of the flat regression line was 14.56 minarc with half the corresponding confidence interval of 19.96 minarc. A reason for this may be the uncertainly in the zero value of the objective fixation disparity that was discussed in Jaschinski [17]: zero is defined by the monocular calibration in each eye, but it seems to be unclear whether this zero value in monocular vision is an appropriate estimation of the supposed zero value in binocular vision that, in principle, remains unknown. Thus a “zero offset” may exist that probably depends on the individual. For this reason, a more precise measure of the prism effect may be the intra-individual difference “prism” minus “no prism”, since the difference eliminates any zero offset. The differences “with prism” minus “without prism” are plotted in Figure 4.

**Figure 5:**
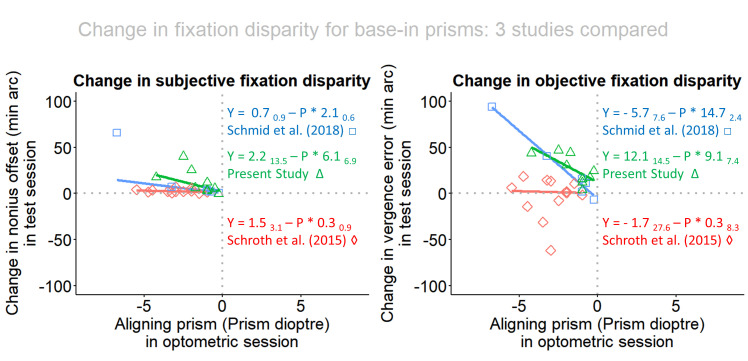
Comparison between the three relevant studies with respect to the prism-effect versus the amount of the aligning prism in base-in cases. Data points, shown as green triangles, represent the present study of one minute prism wearing, blue squares represent Schmid et al. [30] of a few seconds prism wearing and red diamonds refers to Schroth et al. [29] with five weeks prism wearing.

Figure 5 shows an interesting comparison between the three relevant studies with respect to the prism-effect versus the amount of the aligning prism for base-in cases. For oFD, the regression line of the present study with the one-minute prism exposure is practically identical with the one from the Schmid et al. [30] study with a prism expose of 2 – 5 seconds; both studies used the Cross test. This suggests that in the range from a few seconds to 1 minute, the prism effect is very similar.

This result was unexpected given the earlier findings. Schor describes the disparity vergence system to be composed of “fast” and “slow” fusional vergence components [41]. These components operate such in a way that the vergence system adapts with the increasing periods of prism wear. When prism eye glasses are constantly worn, the vergence system may (partly) adapt to this forced-vergence state and the intended reduction in fixation disparity may disappear, or be smaller than intended [37, 49-54]. These earlier findings suggest the conventional view that the present 60 s exposure should have induced smaller prism effects than the 2 – 5 s exposure of Schmid et al. [30]. In actually, they were similar, suggesting that also over a 1 minute prism exposure duration no substantial vergence adaption had occurred. But note that the earlier findings were limited to subjective measures and that the experimental conditions differed, e.g. with respect to the eccentricity, viewing distance, and luminance of the fusion target.

Figure 5 also includes the data points of the study of Schroth et al. [29]: these data points were scattered very much around zero and the correlation was zero. This may be explained by vergence adaptation over the long-term period of prism exposure of 5 weeks. But there was a considerable scatter of data points that has been further analyzed by Schroth et al. [29] in terms of specific individual prism effects, although the group mean effect was zero. Note, however, that a zero change in fixation disparity does not mean that the vergence system is in the same state as without a prism. When applying prisms, the fusional reflex induces a change in the absolute vergence angle in order to compensate the optical effect of the prism. If the fixation disparity with prisms is the same as without prisms, the vergence angle has then changed by an angular amount corresponding exactly to the power of the prism. The resulting vergence state can be advantageous for the following reason: the exo fixation disparity without prisms indicates that the vergence resting position (clinically tested as heterophoria) is more divergent than the vergence stimulus [7]. The corresponding base-in prism induces a more divergent vergence state and therefore shifts the eyes towards their resting position. Experimental evidence for this reasoning comes from clinical studies that showed stable prism corrections and reduced asthenopic complaints when prisms are constantly worn [24, 25]. The advantage of a vergence resting position in terms of a corresponding viewing distance has been shown by Jaschinski [12, 55].

In terms of eye tracker methodology, we conclude that small vergence angles and their changes due to prisms were in the order below 1deg and still could be measured reliably with the present video-based dark pupil eye tracking procedures. This was shown by the fact that the measures of objective fixation disparity were significantly related to the amount of the prism that was applied. These results were achieved with the present purpose-made methods of recording and data analysis that included a control of the pupil size and the correction of the potential artifact on the measured eye position [48, 56]. After the introduction of eye trackers into the domain of fixation disparity research, we today can claim that a complete diagnosis of fixation disparity should cover both subjective and objective measures.

In terms of the physiological vergence eye movement control, we come to the following conclusions:

1. The direction and amount of a subjective aligning prism test such as the Cross test allows for a prediction of the direction and amount the objective FD with an adjusted R-squared of 0.5; for subjective fixation disparity the adjusted R-squared was 0.8.
2. Wearing the aligning prism for 60 sec induced effects in the expected direction and the amount of the changes was linearly related to the amount of the prism, in the objective FD. Only a trend was found for subjective FD. This result refers to base-in prisms in the present study.
3. In terms of prisms adaptation, we conclude that wearing base-in prisms for 2 - 5 sec in Schmid et al. [30] and for 60 sec in the present study showed very similar effects on objective fixation disparity; this suggests that – at least in the present test conditions - no substantial vergence adaptation occurs within one minute. However, there is the caveat that this interpretation is based on the comparison of different groups in two studies. Further research may vary the prism exposure duration as a parameter in an intra-individual experimental design.

## Ethics and Conflict of Interest

The authors declare that the contents of the article are in agreement with the ethics described in http://biblio.unibe.ch/portale/elibrary/BOP/jemr/ethics.html and that there is no conflict of interest regarding the publication of this paper.
